# The Impact of Short-Term Dietary Restriction on Stem Cell Function

**DOI:** 10.1093/geroni/igab046.443

**Published:** 2021-12-17

**Authors:** Archana Unnikrishnan

**Affiliations:** University of Oklahoma, Oklahoma City, Oklahoma, United States

## Abstract

Stem cells play a critical role in the maintenance of tissue function and their proliferative/regenerative capacity is essential to this role. Because stem cells persist over the lifespan of an animal, they are susceptible to gradual accumulation of age-associated damage, resulting in the loss of regenerative function that can impair organ function. Understanding the mechanism(s) that regulates stem cell function is essential for retarding the aging process, and stem cells are attractive targets for aging interventions. Dietary restriction (DR), the most robust anti-aging intervention to-date, has been shown to enhance the activity and integrity of stem cells in a variety of tissues (e.g., muscle, bone marrow, and intestine), and it is believed that effect of DR on stem cells plays an important role in the anti-aging action of DR. For example, DR has been shown to preserve and increase the number of intestinal stem cells (ISCs) and enhance their regenerative capacity in young animals. Data from my lab shows that ISCs from old mice have limited proliferation activity and form few if any organoids in vitro (a surrogate for a fully functional crypt) and that ISCs isolated from old mice on life-long DR show an improved ability to form organoids. While it is well accepted that life-long DR increases lifespan and has anti-aging effects an important aspect of DR that has been largely overlooked is that DR implemented only for a short time early in life can increase lifespan of rodents even when rodents are fed ad libitum the remainder of their life. In line with this, we recently found that ISCs from old mice fed DR for only a short-period resulted in a dramatic increase in ability of the ISCs to form organoids. This is the first evidence that short-term DR administrated late in life can rescue the loss in ISC function that occurs with age.

